# Collagenase-Induced Mouse Model of Osteoarthritis—A Thorough Flow Cytometry Analysis

**DOI:** 10.3390/life12111938

**Published:** 2022-11-21

**Authors:** Blagovesta Boneva, Nikola Ralchev, Petya Ganova, Andrey Tchorbanov, Nikolina Mihaylova

**Affiliations:** 1 Laboratory of Experimental Immunology, Institute of Microbiology, Bulgarian Academy of Sciences, 1113 Sofia, Bulgaria; 2 Laboratory of Immunohistochemistry and Immunopathology, Institute of Microbiology, Bulgarian Academy of Sciences, 1113 Sofia, Bulgaria

**Keywords:** collagenase, experimental osteoarthritis, inflammation, mouse model

## Abstract

Objectives: Osteoarthritis (OA) is a chronic degenerative disorder of the joint characterized by cartilage breakdown and synovial inflammation. A number of different cells of innate and adaptive immunity contribute to joint pathology during OA inflammation. The interaction between the local synovial and systemic inflammatory cellular response and the structural changes in the joint is still unknown. The objective of this study was to investigate the role of the different types of immune cells in the development of OA. Methods: Collagenase-induced osteoarthritis was induced in Balb/c mice; flow cytometry analysis; and histopathological damages were assessed in histological sections stained with H&E, Toluidine blue, and Safranin O. Results: Flow cytometry analysis showed B lymphocyte infiltration in the active phase of inflammation and an increase in the effector T cell population into the synovium. An increased activation state of cytotoxic T cells and of NK cell populations in the spleen and synovium was also found. The differentiation of NK cells from a cytotoxic phenotype in early OA to cells with an effector phenotype in the chronic phase of the disease followed. Conclusions: A number of different cells contribute to inflammatory processes in OA. The correlation between their phenotype and the inflammatory pathophysiology could result in the development of novel approaches to suppress destructive changes in the joint.

## 1. Introduction

Osteoarthritis (OA) is the most common degenerative joint disorder that involves all of the major joint tissues, including articular cartilage, synovium, and subchondral bone [1]. Millions of people all over the world suffer from significant pain, impaired mobility, medical care, and sometimes a loss of independence as a result of OA.

Many pathological changes are observed in the affected joints during OA. These changes include the loss of articular cartilage, chondro/osteophyte formation, and sclerosis of subchondral bone [2,3]. The synovium is also involved in the development of synovitis (the activation of the synovial layer). The formation of osteophytes at the junction of the periosteum and synovium causes obvious negative effects, such as pain and a loss of movement [3]. Thorough research into the pathophysiological mechanisms of OA would contribute to the development of more effective treatments.

Animal models are the most powerful research tools for studying the pathogenesis and potential therapeutic intervention of many different diseases. They are not only important for knowledge development but they also contribute significantly to the “transition” of drug discovery studies toward clinical realization [4,5]. The animal models used in OA research can be divided into induced and spontaneous models [6,7]. Spontaneous models include naturally occurring and genetically based animals. Induced models are further subdivided into surgical and chemical models based on the procedure used to induce OA. Chemical models are generated by the intra-articular injection of modifying factors or by systemically administering noxious agents [4,7,8].

In this study, we used a collagenase-induced experimental OA as a very useful mouse model to study osteoarthritic lesions, osteophyte formation, cartilage erosions, fibrosis, and the activation of synovial macrophages [7,9]. All these important pathological features are also observed in human OA [4]. The development of the studied experimental model can be divided into three phases: the acute phase (from the 1st to the 7th day after intra-articular injection); the active phase (from the 8th to the 25th day after intra-articular injection); and the chronic phase (from the 26th to the 60th day after intra-articular injection). Collagenase mainly attacks joint structures such as tendons and ligaments, which contain collagen type 1, and induces joint instability, showing minimal direct effects on joint cartilage [7,10]. The pathological processes during the collagenase induction of OA could be separated in two stages: the first stage represents the induction of joint instability as a result of the collagenase injection, and the second stage exhibits the development of osteoarthritic lesions. Induced inflammation in the second stage of OA leads to the release of proinflammatory factors and mediators in joint fluid, as well as the attraction of different cell types into the articular cavity.

The pathology of osteoarthritis involves activating the immune system and causing inflammation in which monocytes, neutrophils, lymphocytes, and platelets play an important role. The balance of the immune response and the neutrophil–lymphocyte ratio, monocyte–lymphocyte ratio, and platelet–lymphocyte ratio, as predictors of many inflammatory and immune diseases, was determined [11,12,13,14].

However, there is a lack of accurate information on the role of immune cells and how they affect OA joint remodeling. Lymphocytes and monocytes are key players in innate and acquired immunity, and the monocyte–lymphocyte ratio represents the balance and degree of progression of immune disease. Jiang et al. showed in their study that the number of neutrophils and monocytes in the blood of patients with osteoarthritis was higher compared to the healthy control group, while the number of lymphocytes and platelets was lower [12]. The observed increase in the number of monocytes may be caused by the progression of inflammation. Monocytes can be activated via inflammasome-mediated pathways, and chronic low-grade inflammasome activation contributes to drive OA progression [1,12]. During local inflammation, a decrease in the number of lymphocytes was observed, which may be related to their accumulation at the site of inflammation [11,14]. Notably, Du et al. found that the lymphocytes–monocytes ratio in rheumatoid arthritis patients was higher than those with OA and healthy controls [11]. A better understanding of the inflammatory pathophysiology of OA could result in the development of novel approaches to suppress destructive changes in the joint and prevent permanent functional impairment.

To investigate how the diversity of immune cells may contribute to the pathogenesis of osteoarthritis, we characterized, phenotypically, synovial tissue, bone marrow, and splenic cell isolates and analyzed how their phenotypes correlate to the developing phases of a collagenase-induced model of OA.

## 2. Materials and Methods

### 2.1. Mice

Female Balb/c mice were obtained from The Jackson Laboratory (Bar Harbor, ME, USA) and maintained in our animal facility under specific-pathogen-free (SPF) conditions. Groups of 10–12-week-old animals were randomly assigned to the respective groups (five animals per group). The animals were housed with food and water ad libitum and maintained on 12 h light cycles in barrier-type animal house under specific-pathogen-free (SPF) conditions. All animal manipulations and study protocols were approved by the Animal Care Commission at the Bulgarian Food Safety Agency (BFSA) (License #306) and were conducted in accordance with the International regulations (EU Directive 2010/63/EU) for the Care and Use of Laboratory Animals.

### 2.2. Antibodies

Anti-mouse fluorescein isothiocyanate (FITC)-conjugated CD4 (clone GK1.5), CD8 (clone 53-5.7), CD11c (clone N418), and CD335 (clone 29A1.4); eFlour450-conjugated CD3 (clone 145-2C11); phycoerythrin (PE)-conjugated F4/80 (clone 145-BM8), CD69 (clone H1.2F3), CD19 (clone 6D5), and CD 107a (LAMP-1) (clone 1D4B); Alexa Flour 700-conjugated Ly6C (clone HK1.4); allophycocyanin (APC)-conjugated CD4 (clone GK1.5), CD25 (clone PC61), CD206 (clone C068C2), and CD11b (clone M1/70); Pacific blue-conjugated Ly6G (clone 1A8) and CD80 (clone 16-10A1) (BioLegend, Amsterdam, The Netherlands) were used for fluorescence-activated cell sorting (FACS) experiments. Respective isotype-matched IgG controls (BioLegend) were used for compensation and confirmation of antibody specificity.

### 2.3. Induction of Osteoarthritis

The animals were anesthetized and injected intra-articularly through the patellar ligament in both knees with 10 μL of collagenase solution (from *Clostridium histolyticum* type IA, Sigma-Aldrich, Darmstadt, Germany, #C9891) in a concentration of 2 mg/mL in TRIS-HCl, pH 7.0 (0.005 M Tris-hydrochloride, 0.005 M CaCl_2_). A control group of animals was injected intra-articularly with 10 μL of PBS (phosphate-buffered saline, pH 7.4)/per knee.

### 2.4. Isolation of Cells from Spleen and Synovium

On the 7th, 14th, and 30th day after intra-articular injection, the spleens were taken from sacrificed collagenase-injected animals and from healthy controls, and monocellular suspensions were prepared by grinding through sterile cell strainers (BD Biosciences, Erenbodegem, Belgium). Erythrocytes were lysed with hypotonic ammonium RBC lysis buffer (150 mM NH_4_Cl, 10 mM KHCO_3_, 0.1 mM Na_2_EDTA, pH 7.2), and the splenocytes were washed twice with PBS containing 2.5% FCS (fetal calf serum) and 0.05% sodium azide. The lymphocytes were counted and used for flow cytometry analyses.

### 2.5. Synovial Extracts

Patellae with surrounding soft tissue (tendon and synovium) were excised from collagenase-injected animals (on the 7th, 14th, and 30th day after intra-articular injection) and from healthy controls and were incubated in 200 μL of serum-free RPMI (Roswell Park Memorial Institute, Buffalo, NY, USA) 1640 medium (HiMedia Laboratories GmbH, Einhausen, Germany) for 2 h at 37 °C, as described by van de Loo et al. [15]. The supernatants were collected and centrifuged at 1200× *g* for 10 min. The isolated pool of cells was counted and used for flow cytometry analyses.

### 2.6. Isolation of Cells from Bone Marrow (BM)

The femurs and tibias were isolated from the mice under sterile conditions and were placed into a sterile cell culture dish. The muscles and residue tissues surrounding the femur and tibia were removed with sterile forceps and scissors. Both ends of the bones were cut by using a 23-gauge needle and a 10 mL syringe filled with ice-cold PBS; the bone marrow was flushed out onto a 70 μm nylon cell strainer placed in a 50 mL conical tube. The bone marrow was smashed through the cell strainer with a plunger, and the cell suspension was centrifuged at 1300 rpm for 10 min at 4 °C. The cell pellet was resuspended with 1 mL RBC lysis buffer. After incubation for 2–3 min at RT, the cells were centrifuged at 1300 rpm for 10 min at 4 °C and prepared for flow cytometry analyses, as described above.

### 2.7. Flow Cytometry Analysis

The isolated cells (1 × 10^6^ cells per mL) from spleen, BM, and synovium were washed with PBS (containing 2.5% FCS and 0.05% sodium azide) and incubated with one of the following mixes of anti-mouse antibodies: for B cell gating—CD19 antibody; for T cell gating—CD3, CD4, and CD8 antibody mixture; for activated effector T cell gating—CD3, CD4, CD25, and CD69 antibody mixture; for activated cytotoxic T cell gating—CD3, CD8, and CD107a antibody mixture; for NK cell gating—CD3 and CD335 antibody mixture; for activated NK cell gating—CD3, CD335, and CD107a and CD3, CD335, CD11b, and CD27 antibody mixtures; for macrophages gating—F4/80, CD11b, CD80, and CD206 antibody mixture; for neutrophils gating—CD11b and Ly6G antibody mixture; for DC gating—CD11b and CD11c antibody mixture; for eosinophils gating—SSC parameter and CD11b antibody; for monocytes gating—SSC parameter and CD11b and Ly6C antibody mixture. Each incubation step was performed for 20 min on ice (2 × 10^5^ cells/tube). Thirty thousand cells were collected and analyzed from each sample with a BD LSR II flow cytometer using Diva 6.1.1. software (BD Biosciences, San Jose, CA, USA).

### 2.8. Histological Analysis

Groups of mice (five animals each) injected with collagenase IA type were sacrificed by cervical dislocation 7, 14, and 30 days after intra-articular injection. Dissected knee joints were fixed in 4% phosphate-buffered formalin (pH 7.4) for 5 days and, subsequently, decalcified in 20% EDTA for 10 days. After washing, the knee joints were embedded in paraffin (Paraplast Plus^®^, Sigma-Aldrich). Sections (5–7 mm) were deparaffinized in xylene substitute (Tissue-Tek ^®^ Xylene, Sakura Finetek, Torrance, CA, USA) followed by dehydration in a graded series of ethanol to water. Hematoxylin and eosin (H&E) staining was performed to observe the general morphology of tissues. Staining with Toluidine blue and Safranin O with fast green counterstain was performed to analyze articular cartilage and evaluate the presence of proteoglycans and glycosaminoglycans.

### 2.9. Statistical Analysis

All statistical analyses were performed with Prism software from GraphPad (San Diego, CA, USA). Two-way ANOVA test was used to determine differences between each two groups, and values in the figures correspond to mean ± SD. A value of *p* < 0.05 was considered to be statistically significant.

## 3. Results

### 3.1. Histopathological Analysis of the Experimental Mouse Model of OA during the 7th, 14th, and 30th Day of Development

**Figure 1:**
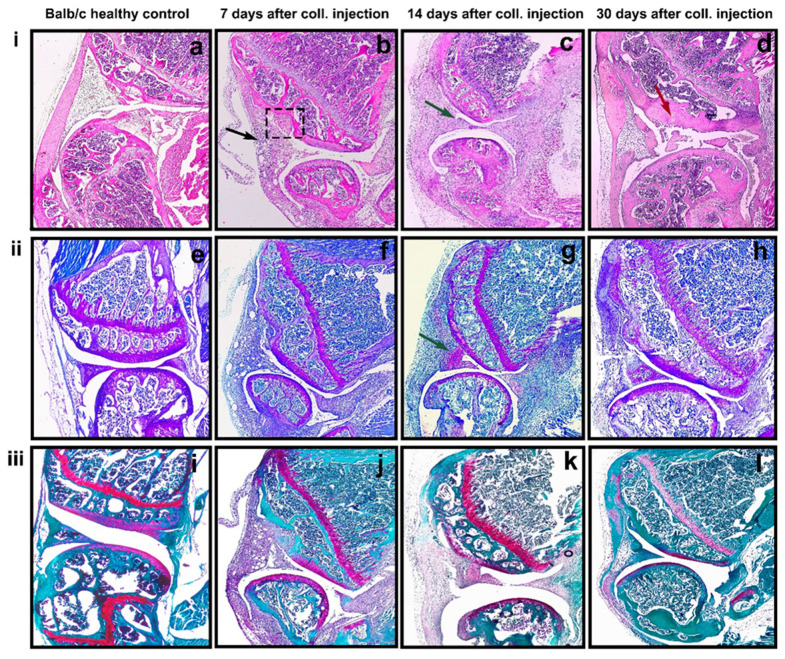
Histologic features of knee joint after intra-articular injection of collagenase type IA. (**i**(**a**–**d**)) H&E section; (**ii**(**e**–**h**)) Toluidine blue staining section; (**iii**(**i**–**l**)) Safranin O staining section. Cell infiltration of the synovium—black arrow; partial destruction of the cartilage—dotted rectangle; pannus formation—green arrow cartilage; and subchondral bone destruction—red arrow. (Original magnification ×40).

Dislocation of the patella was observed 7 days after the intra-articular injection of collagenase type I, while the same was not found in the control knee joints. Basic histological staining by hematoxylin and eosin was used for the general assessment of cell and tissue morphology and distribution. At day seven, mild mononuclear cell infiltration into the synovium was visible (Figure 1i(b)—black arrow). The partial destruction of cartilage tissue and invasion into the synovium was detected also at day seven (Figure 1i(b)—dotted rectangle). Toluidine staining showed the reduction of cartilage content at the early stage of inflammation (Figure 1ii(f)), and with disease progression, the synovial tissue became hypercellular and well vascularized. At day 14, massive inflammation of the synovium with pannus formation was observed (Figure 1i(c)—green arrow). Pronounced depletion of Safranin O staining was clearly visible (Figure 1iii(k)). At day 30, severe GAGs (glycosaminoglycans) and PGs (proteoglycans) loss in cartilage were observed (Figure 1iii(l)). Mild cartilage and subchondral bone destruction were also found with H&E staining (Figure 1i(d)—red arrow).

### 3.2. Distribution of B, T, and NK Cells in the Synovium, BM, and Spleen during the Experimental OA Development

**Figure 2:**
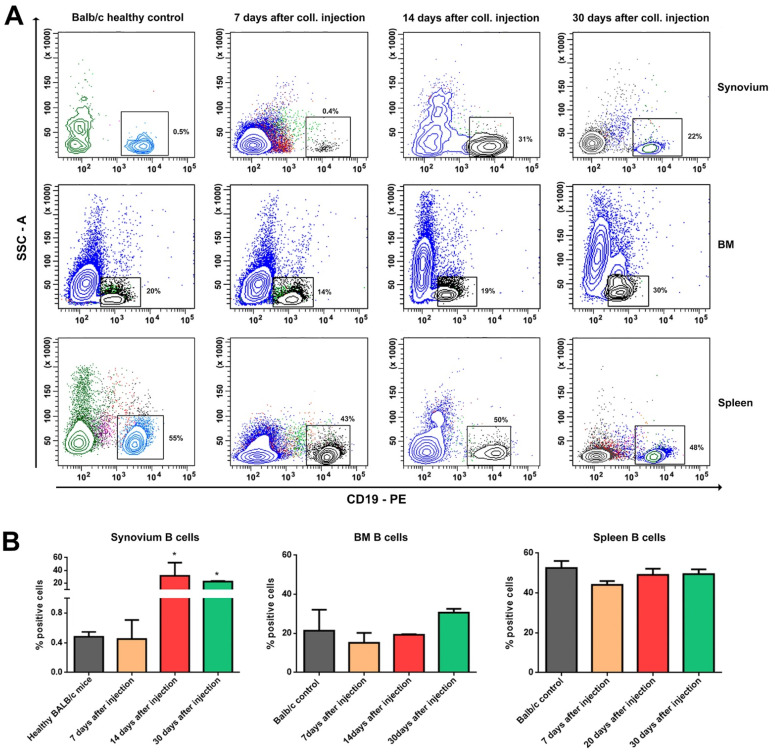
Distribution of B cells in synovium, BM, and spleen during OA development. (**A**) Flow cytometry analysis of the gated B cell population in the synovium, BM, and spleen in mice on the 7th, 14th, and 30th day of OA development. Ten thousand cells were analyzed from each sample. Data are representative of at least 5 experiments. (**B**) The extracted results from all experiments are presented graphically. Results are represented as mean SD (n = 5). Data were analyzed by two-way ANOVA test followed by Bonferroni’s multiple comparison test. * *p* < 0.05.

Under inflammatory conditions such as collagenase-induced osteoarthritis, many different cell types are recruited to the affected joint. In the current study, we have analyzed the proportions of B and T cells in three main compartments: the synovium, BM, and spleen during the different periods of OA development (on the 7th, 14th, and 30th day post collagenase injection). No significant increase was found in the percentage of B cells in the synovium on the seventh day after the collagenase injection compared to the healthy Balb/c controls. On the 14th and 30th day after OA induction, the percentage of B cells in the synovium increased significantly compared to the control group. Results from BM and the spleen showed a weak and not statistically significant decrease during the same period (Figure 2).

**Figure 3:**
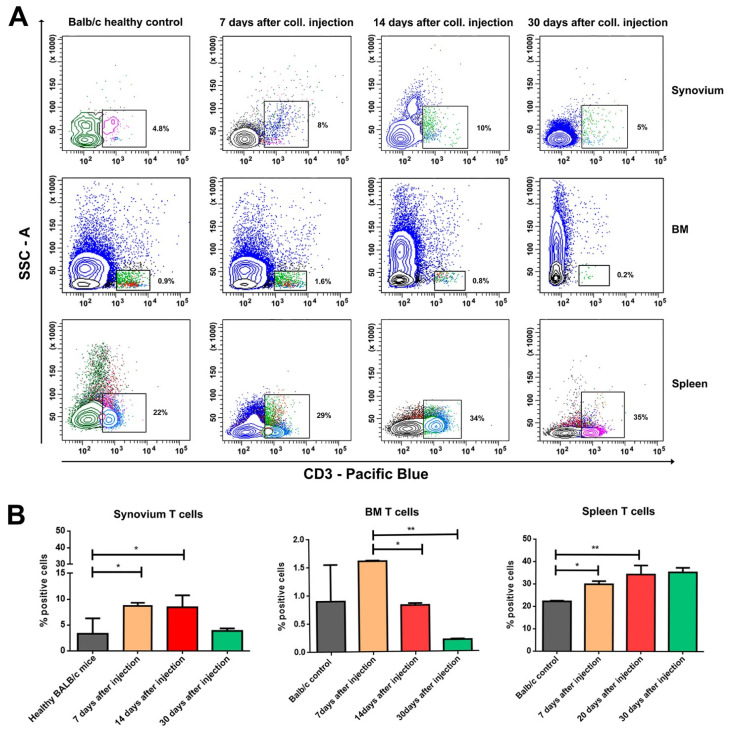
Distribution of T cells in synovium, BM, and spleen during OA development. (**A**) Flow cytometry analysis of the gated T cell population in the synovium, BM, and spleen in mice on the 7th, 14th, and 30th day of OA development. Ten thousand cells were analyzed from each sample. Data are representative of at least 5 experiments. (**B**) The extracted results from all experiments are presented graphically. Results are represented as mean SD (n = 5). Data were analyzed by two-way ANOVA test followed by Bonferroni’s multiple comparison test. * *p* < 0.05 and ** *p* < 0.01.

**Figure 4:**
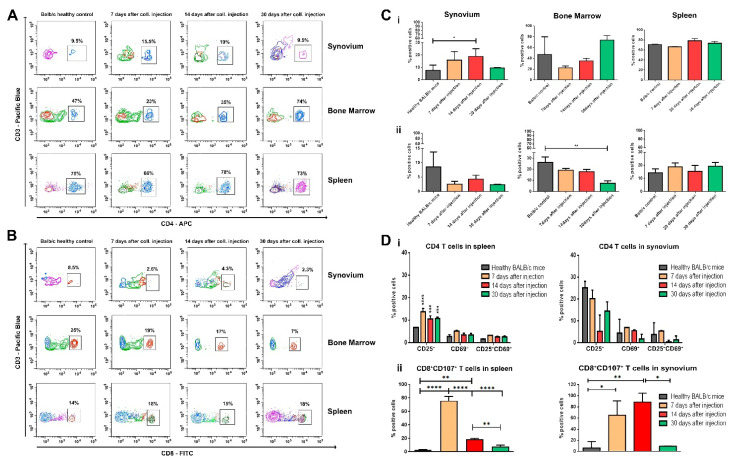
Flow cytometry analysis of the gated CD3^+^CD4^+^ (**A**) and CD3^+^CD8^+^ (**B**) cell population in the synovium, BM, and spleen in mice on the 7th, 14th, and 30th day of OA development. Ten thousand cells were analyzed from each sample. Data are representative of at least 5 experiments. (**C**) (**i**) The extracted results for CD3CD4 double-positive cells from all experiments are presented graphically. (**ii**) The extracted results for CD3CD8 double-positive cells from all experiments are presented graphically. (**D**) The state of T cell activation in the synovium and spleen during OA development: (**i**) The extracted results of activated CD3^+^CD4^+^ T cells from all experiments are presented graphically. (**ii**) The extracted results of activated CD3^+^CD8^+^ T cells from all experiments are presented graphically. Ten thousand cells were analyzed from each sample. Results are represented as mean SD (n = 5). Data were analyzed by two-way ANOVA test followed by Bonferroni’s multiple comparison test. * *p* < 0.05, ** *p* < 0.01, *** *p* < 0.001, and **** *p* < 0.0001.

An increased population of CD3-positive cells was found in the synovium, BM, and spleen after the induction of inflammation (Figure 3). Through analyzing the population of T helpers and cytotoxic T cells in the targeted compartments (Figure 4A,B), we observed that the percentage of CD3^+^CD4^+^ T cells in the synovium increased, while the number of CD3^+^CD8^+^ T cells shrank during the observation (Figure 4C(i,ii), left column). The percentage of helper T cells in BM decreased in the acute phase of the disease, but it was elevated at the end of the observation period (Figure 4C(i), middle column). We observed the opposite trend for the number of cytotoxic CD8^+^ T cells in the BM compartment (Figure 4C(ii), middle column). There was no difference in both T cell subpopulations in the spleen compartment (Figure 4C, right column).

The levels of T cell activation were also analyzed by tracing out the expression of CD25 and CD69 markers. The results have shown that although there was no difference in the population of splenic CD3^+^CD4^+^ T cells compared to the healthy controls (Figure 4C(i), right panel), the mice with induced OA had significantly more activated helper cells. This was shown by the elevated expression of the late activated marker CD25^+^ (Figure 4D(i), left graph). Such elevated levels of activation were not found in the population of synovium CD4^+^ T cells (Figure 4D(i), right graph).

As a marker for the activation of cytotoxic T cells, we explored the cell surface expression of CD107a (LAMP-1). An increased expression level of CD107a in OA groups was found despite the decreased number of CD3^+^CD8^+^ T cells in the synovium. The elevated levels of activation were reported on the 7th and 14th day after OA induction (Figure 4D(ii), right graph).

The highest activation value of splenic CD8^+^ T cells was measured on the seventh day of the collagenase injection (Figure 4D(ii), left graph).

**Figure 5:**
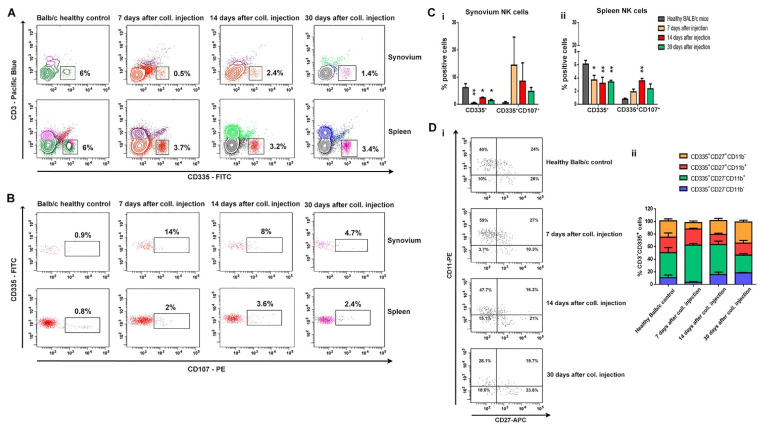
Distribution and activation state of NK cells in synovium, BM and spleen during OA development. Flow cytometry analysis of the gated CD3^−^CD335^+^ NK (**A**) and CD3^−^CD335^+^ CD 107^+^ NK (**B**) cell populations in the synovium and spleen in mice on the 7th, 14th, and 30th day of OA development. Ten thousand cells were analyzed from each sample. Data are representative of at least 5 experiments. (**C**) The extracted results for CD3^−^CD335^+^ cells (**i**) and for CD3^−^CD335^+^CD107^+^ cells (**ii**) from all experiments are presented graphically. (**D**) (**i**) Flow cytometry analysis of the gated CD3^−^CD335^+^ NK cell population in BM in mice on the 7th, 14th, and 30th day of OA development. (**ii**) The extracted results for CD3^−^CD335^+^ CD11b^+/−^CD27^+/−^ cells from all experiments are presented graphically. Results are represented as mean SD (n = 5). Data were analyzed by two-way ANOVA test followed by Bonferroni’s multiple comparison test. * *p* < 0.05, ** *p* < 0.01.

The population of CD3^−^ CD335^+^ NK cells was also analyzed (Figure 5). The results showed that the population of NK cells in the synovium and spleen of collagenase-injected animals was significantly smaller than the one of the healthy control group (Figure 5A,C). Surprisingly, this small population was very activated due to the high surface expression of CD107 (Figure 5B,C).

To follow NK cell distribution in BM by the surface expression of specific markers, we discriminated four NK subsets: CD335^+^CD27^−^CD11b^−^, CD335^+^CD27^+^CD11b^−^, CD335^+^CD27^+^CD11b^+^, and CD335^+^CD27^−^CD11b^+^ (Figure 5D). We found that CD11b and CD27 NK cell profiles changed dramatically during OA development. While on the 7th day after OA induction, the predominant subset of NK cells was with the phenotype of cytotoxic NK cells (CD335^+^CD27^−^CD11b^+^); on the 30th day after collagenase injection, the subset of NK cells was mostly with an effector phenotype (CD335^+^CD27^+^CD11b^−^) (Figure 5D(ii)).

### 3.3. Distribution of Myeloid Cells in the Synovium and BM during OA Development

After the collagenase injection, the products of cartilage breakdown are released into the synovial fluid and are phagocytosed by synovial cells. This leads to the expansion of synovial inflammation amplified by activated synovial resident cells and infiltrating immune cells.

The role of myeloid cells in the pathophysiology of OA is still debatable, and serious efforts and investigations are focused on this field. Macrophages are a highly heterogenous group of cells, which can quickly change their function and phenotype in response to local microenvironmental signals. They play a critical role in OA pathogenesis through the induction of inflammatory mediators, growth factors, and proteinases.

**Figure 6:**
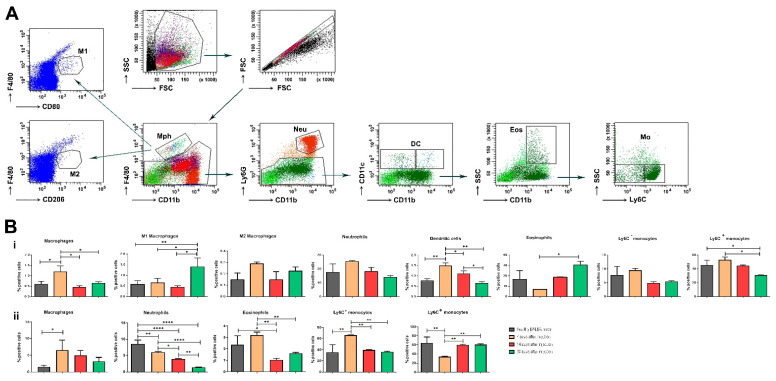
Distribution of myeloid cells in synovium and BM during OA development. (**A**) Flow cytometry analysis and gating strategy of myeloid cell populations in the synovium and BM on the 7th, 14th, and 30th day of OA development. (**B**) (**i**) The extracted results for myeloid cells in BM from all experiments are presented graphically. (**ii**) The extracted results for myeloid cells in the synovium from all experiments are presented graphically. Data are represented as mean SD (n = 5). Results were analyzed by two-way ANOVA test followed by Bonferroni’s multiple comparison test. * *p* < 0.05, ** *p* < 0.01, **** *p* < 0.0001.

In this study, we traced F4/80-positive macrophages in the BM of osteoarthritic animals (Figure 6A). As expected, seven days after the intra-articular injection of collagenase at the acute phase of OA, the percentage of macrophages increased (Figure 6B(i)). Next, we defined the M1/M2 macrophage phenotype, and a slight non-significant increase in the M2 subtype was monitored at the acute phase of OA development with predominant polarization to M1 macrophages in the chronic phase of the disease (Figure 6B(i)).

Normally, resident macrophages in the synovium are responsible for the support of joint homeostasis and the communication between different cell types. Under inflammatory conditions, such as collagenase-induced osteoarthritis, monocytes are the cell type that have a very complex role in this process. After the differentiation of monocytes from hematopoietic stem cells, two functionally distinct monocyte subsets are described: proinflammatory Ly6C^high^ and patrolling Ly6C^low^ monocytes. At the site of injury, monocytes may differentiate into M1-like proinflammatory or M2-like anti-inflammatory macrophages, depending on the environmental cues. In our model, we observed a high percentage of Ly6C^low^ monocytes attracted to the synovium (Figure 6B(ii)). This is related to the higher percentage of Ly6C^high^ monocytes that reside in BM and, upon release from BM into circulation, can transform into Ly6C^low^ monocytes (Figure 6B(i)).

It has already been shown that neutrophils are also involved in OA development [16] and interact with NK cells [17]. Here, we can confirm the increase in the neutrophil population seven days after OA induction in BM (Figure 6B(i)), while in the synovium, the neutrophil population decreased during the different stages of OA development (Figure 6B(ii)).

Little is known about the role of dendritic cells (DC) in OA immunopathogenesis. Their peripheral tolerance potential and the possibility to become regulatory cells under specific circumstances could be used as an instrument to eliminate immunoinflammatory manifestations in OA. We analyzed the population of CD11b^+^ CD11c^+^ cells and found an increased percentage of cells expressing these markers in BM on the seventh day of OA induction (Figure 6B(i)). We did not detect the same cell population in the synovium.

Eosinophils are an atypical cell population in the collagenase-induced model of OA. A limited number of studies are available for the discussion of eosinophils significance in the pathogenesis of OA. In our study, seven days after OA induction, the eosinophil population was elevated in the synovium, followed by a reduction towards the end of the observation (Figure 6B(ii)). At the same time, the exact opposite trend was found in BM (Figure 6B(i)).

## 4. Discussion

Osteoarthritis (OA) is the most common form of arthritis and one of the leading causes of disability worldwide. According to the World Health Organization, 343 million people are affected globally [18]. OA is a chronic degenerative disorder of the joint that is characterized by cartilage breakdown and synovial inflammation. It is a multifactorial disorder, and no single etiological mechanism has been found common among all forms of the disease. Although OA is primarily associated with aging, there are other important contributing factors. These include genetics, underlying anatomical and orthopedic disorders (i.e., congenital hip dislocation), obesity, underlying inherited or acquired metabolic disease, endocrine disease, various disorders of bone turnover and blood clotting, joint infection, crystal deposition, previous rheumatoid arthritis (RA) or a history of joint trauma, muscle weakness, or joint instability [19].

In the current study, we used a collagenase-induced mouse model of OA as a model that reproduces some of the main symptoms associated with OA onset and development in humans. Clarifying the exact role of the different cell types of innate and acquired immunity, as well as the interactions and communication between the cells, would transfer this knowledge to human OA in order to explain the pathophysiology of the disease.

The clinical symptoms of OA are joint swelling, synovitis, and inflammatory pain. A key factor in OA pathophysiology is synovitis, which is directly responsible for several clinical symptoms and reflects the structural progression of the disease by the action of several soluble mediators [20]. Different cell types usually participate in immunological processes in OA as bystanders and as actors. The products of cartilage breakdown that are released into the synovial fluid are phagocytosed by synovial cells, thereby amplifying synovial inflammation. In turn, activated synovial cells in the inflamed synovium produce proinflammatory mediators that lead to excess production of the proteolytic enzymes responsible for cartilage breakdown, creating a positive feedback loop. The inflammatory response is amplified by activated synovial T cells, B cells, and infiltrating macrophages. To counteract this inflammatory response, the synovium and cartilage may produce anti-inflammatory cytokines.

B cells are rarely found in the OA synovial membranes, but those that are present exhibit an activated state [21]. Antibodies against autoantigens, such as the breakdown products of type I and type II collagen, have been identified in OA cartilage, suggesting that these antibodies are locally produced by synovium infiltrating B cells [22]. Our results confirmed the previous studies reporting infiltration of B lymphocytes after the acute phase of the inflammation. The degree of B cell infiltration is directly correlated with the severity of the local inflammation. It is still an open discussion whether the generation of antibodies against self-structures such as cartilage degradation products is a primary or secondary immune response. Moreover, autoantibodies against cartilage and bone proteins, such as the cartilage intermediate layer protein [22], and YKL-39 [23], which have been found in the serum of RA patients, have also been detected in OA patients. The release of different osteoarthritic antigens may drive chronic B cell autoimmunity, which could in turn positively or negatively modulate the disease process [23,24].

CD4^+^ T cells are crucial for directing appropriate immune responses during the host defense and for the pathogenesis of inflammatory diseases. Guerassimov, A. et al. showed that T cell responses to cartilage matrix proteins are higher in OA patients than in healthy donors [25]. Accordingly, we found increased proportion of CD3^+^CD4^+^ T lymphocytes in the synovium of the experimental animals. Other studies have already shown that in the early OA predominantly CD4^+^ T lymphocytes with prevalent Th1 cell polarization are infiltrated [26,27]. It was shown that CD3^+^CD4^+^ cells are the most prevalent type of activated inflammatory cells in the synovium during OA. We showed the presence of high activated splenic CD4^+^ T cells early in OA development compared to the resident ones in the synovium. On the other hand, the percentage of the CD3^+^CD8^+^ T cells was significantly reduced compared to the control group, but the activation status of these cells was very elevated in respect of the expression of CD107a. Recently, CD107a (lysosomal-associated membrane protein-1 or LAMP-1) has been described as a marker of CD8^+^ T-cell degranulation following stimulation [28]. Additional research is needed to define the exact role of CD8+ T-cells in the pathogenesis of collagenase-induced OA.

The main role of NK cells is the elimination of viral-infected and transformed cells and the secretion of proinflammatory cytokines. There are data that suggest NK populations enriched the inflamed joints of patients with various arthritic diseases, including RA patients [29,30,31]. NK cells are presented in the synovial fluids of RA patients and are considered to be important players in bone destruction. The role of NK cells in OA pathology needs additional research. Huss et al. showed that NK cells comprised nearly 30% of the CD45^+^ mononuclear cell infiltrate in synovial tissues from OA patients [32]. In our study, we observed the opposite tendency, which can be defined by the nature of the model. Although we reported a decreased proportion of NK cells in the spleen and, locally, in the synovium, the cells showed a high activation state concerning the expression of CD107a. Employing CD107a as a marker of NK cell functional activity allows the identification of a large fraction of activated NK cells that degranulate in the absence of cytokine secretion. We can speculate whether these are resident cells and not infiltrated ones. In a more in-depth phenotype analysis of NK cells in bone marrow, we found a change in cell differentiation from a cytotoxic phenotype (CD335^+^CD27^−^CD11b^+^) in the early OA to cells with an effector phenotype (CD335^+^CD27^+^CD11b^−^) in the chronic phase of the disease. With the exception of NK cell infiltration, our data confirmed the results of Jaime et al. that suggests NK cells from peripheral blood exhibited a clear cytotoxic function, but in the synovial fluid from OA patients presented an immunoregulatory-like phenotype [33].

Some functional units of the innate immune system—monocytes and macrophages—are also involved in the pathogenesis of OA. Monocytes and macrophages are heterogeneous cell populations displaying a substantial degree of plasticity. Proteins from OA synovial fluid can induce macrophages to produce inflammatory cytokines via the TLR-4 signaling pathway. Interestingly, recent data suggest that these events may occur early in the disease, so innate immunity may be a driver of the OA process. Synovial fluid from patients with early OA cartilage damage showed increased fibroblast-like synoviocyte responses to TLR-2 and TLR-4 ligands. It is already known that macrophages developed from fetal progenitors may stay in the adult tissues. Additionally, macrophages can self-maintain independently of monocytes [32]. It seems that macrophages found in the healthy synovium are predominantly monocyte-independent, and their contribution to protect the joint homeostasis includes the formation of a barrier and clearance from debris [34]. We traced the activation state of terminally differentiated macrophages based on microenvironmental signals. As expected, at the acute phase of the disease, the number of macrophages was higher compared to the healthy control.

Our results demonstrated that non-classical Ly6C^−^ monocytes differentiate into inflammatory macrophages (M1) during arthritis development, and these cells are responsible for disease pathogenesis. The data suggest that circulating Ly6C^−^ monocytes recruited to the joint upon injury may manage the development and resolution of joint inflammation.

Eosinophils are multifunctional granulocytes considered as the effector cells involved in helminth infection and allergic diseases. The cells participate in the regulation of adaptive immune responses, especially in inflammatory and autoimmune disorders. In regard to the role of eosinophils in the pathology and development of OA, additional studies are needed. It is also known that during acute inflammation, polymorphonuclear neutrophils migrate into tissues as an earlier event, followed by the recruitment of monocytes that further differentiate into tissue macrophages [35]. Wang et al. have shown that neutrophils are the major source of cartilage-degrading enzymes in a murine model of early OA [36].

In general, many conflicting results have been reported regarding cellular inflammation in OA. A number of different cell types contribute to inflammatory processes. Possible reasons for these diverging results might be heterogenicity regarding inflammatory activity and the different experimental setups.

Flow cytometric analysis of mice’ synovium on the 7th, 14th, and 30th day after collagenase injection could be addressed by histological findings, i.e., higher mononuclear cell infiltration (monocytes, macrophages, and CD4 T cells) corresponds to the synovial inflammation (synovitis) and newly formed blood vessels seen in the histological samples during early OA. Synovitis and its symptoms are present before bone changes have occurred and, thus, synovial inflammation has much greater significance in the early phase of the disease due to joint instability and initial cartilage lesions. This leads to the production of proinflammatory cytokines and mediators of joint damage. As shown in this study, the synovial membrane in early OA exhibited synovial lining cell thickness, the vascularity and expression of inflammatory cells and mediators, determined a higher hyperplastic and inflammatory component of the synovitis at that stage. It was confirmed by other authors that synovitis could be the risk factor for OA progression. At a later stage of OA development, the calcified cartilage layer vanishes, and the bone plate thickens substantially at the expense of fatty bone marrow.

The chronic nature of OA, the variability in the onset of symptoms, and the rate of progression in humans present challenges for clinical studies. Understanding the pathogenesis of OA and its multifactorial etiology, using induced animal models as powerful research tools, provides the opportunity to develop novel and effective treatments, which are currently lacking.

## Data Availability

Data is contained within the article.
